# Intracerebral Schwannoma in a 16-Year-Old Girl: A Case Report and Review of the Literature

**DOI:** 10.1155/2013/171494

**Published:** 2013-09-25

**Authors:** R. Srinivas, D. Krupashankar, V. Shasi

**Affiliations:** ^1^Department of Neurosurgery, Sri Ramachandra Medical Centre and Research Institute, Chennai, Tamil Nadu 600116, India; ^2^Department of Pathology, Government Medical College, Thrissur, Kerala 680596, India

## Abstract

Intracerebral schwannomas are rare tumors of the CNS. We report a rare case of intracerebral schwannoma, presenting as a cystic and solid frontoparietal mass, arising in a 16-year-old girl. The patient presented seizures and headache. Neuroradiologic findings showed a left frontoparietal lesion with cystic and tissular components. The tumor was removed through a left frontoparietal craniotomy. Histological features confirmed the diagnosis of intracerebral schwannoma. Intracerebral schwannomas, unrelated to cranial nerves, are rare. The Schwann cells are not indigenous to brain substance, and hence histogenesis of these tumours has attracted a lot of speculation, but because most reported cases have involved young patients, a developmental origin has been suggested. The theories and literature related to this case are reviewed.

## 1. Introduction

 Intraparenchymatous schwannomas of the central nervous system are rare. A literature survey revealed reports of 65 such cases [1]. The presence of a cyst together with the tumor appears to be characteristic of such intraparenchymal schwannomas of the brain [2]. Here we report one such rare occurrence in a 16-year-old female with review of the literature.

## 2. Case History

**Figure 1:**
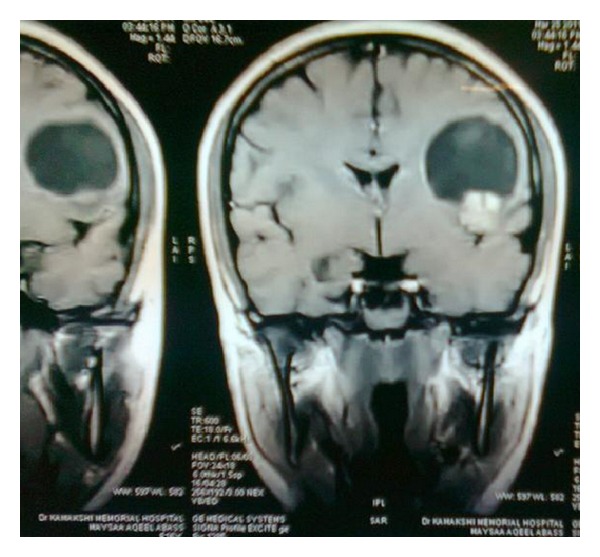
Plain MRI—coronal section showing lesion in the left frontoparietal region.

**Figure 2:**
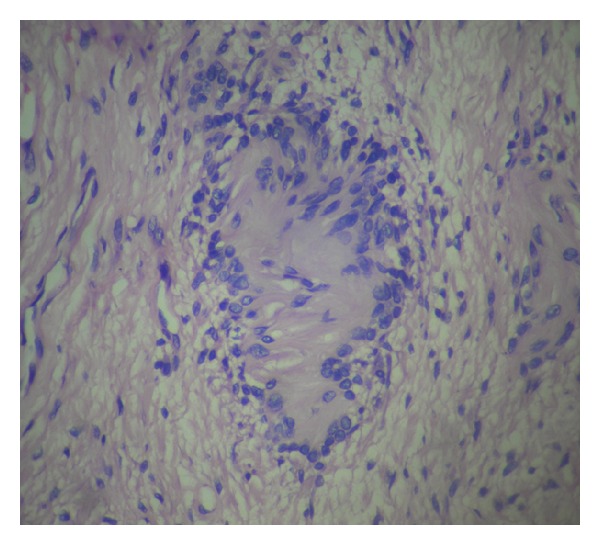
H&E, 400x; Verocay body in Antoni A areas.

**Figure 3:**
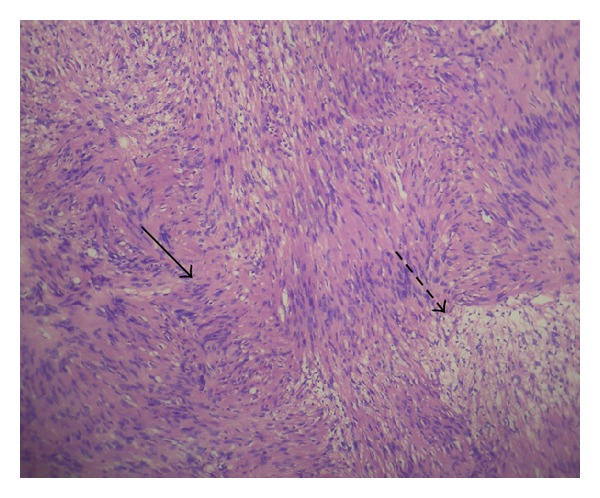
H&E, 100x; Arrow—palisading spindle cells; dotted arrow—collection of foam cells.

 A 16-year-old female was admitted with headache and one episode of seizure. Neurological examination was unremarkable, and cognitive functions were normal. MRI brain showed intracerebral space occupying lesion in the left frontoparietal lobe (Figure 1). Preoperative diagnosis was cystic glioma. The patient was operated under general anesthesia. She underwent left frontoparietal craniotomy, and tapping of cyst along with excision of nodular portion was done. On microscopic examination, there were hypercellular and hypocellular areas. The hypercellular areas were comprised of fascicles of slender elongated spindle cells with wavy serpentine nuclei and formation of Verocay bodies (Figure 2) in some areas (Antoni A). Other areas were hypocellular with myxoid background and occasional foamy macrophages (Antoni B) (Figure 3). Reticulin stain showed a rich pericellular reticulin staining in Antoni B areas. The tumor was then diagnosed as Intracerebral schwannoma. The postoperative period was uneventful, with the patient remaining free of disease after two-years follow-up period.

## 3. Discussion

 Schwannomas are benign tumors accounting for approximately 8% of all intracranial lesions [2]. Intracranial schwannomas not arising from the facial, trigeminal, or vestibular nerves are extremely rare in non-neurofibromatosis patients [3]. Only few well-documented cases of intracerebral schwannomas have been reported in the world literature [4]. 

 Schwannomas commonly arise from the nerve sheaths of peripheral and cranial nerves. Thus, since the central nervous system is devoid of the Schwann cells present in nerves, the pathogenesis of intracerebral schwannomas is unclear. Several theories have been proposed for their intracerebral occurrence. These theories can broadly be considered in two groups, the developmental and nondevelopmental. According to the developmental theory, aberrant Schwann cells in the brain parenchyma may occur due to the transformation of the mesenchymal pial cells [5] or from displaced neural crest cells that form foci of the Schwann cells (“schwannosis”) [6]. The relatively young age at presentation also suggests a developmental etiology. Nondevelopmental theories base their assumption on the fact that the Schwann cells are present within the perivascular nerve plexuses and the large arteries in the subarachnoid spaces [7], although the existence of these structures deep in the brain parenchyma is doubted [8]. However, the Schwann cells are present in the adrenergic nerve fibers innervating the cerebral arterioles [9]. These nerve plexi are common in tela choroidea, which may explain their predilection for periventricular location [10].

 Intraparenchymal schwannomas are detected either in the first two decades, when they present with an indolent, slow-growing course, or in the elderly, when their symptoms evolve rapidly [4]. Males are affected more often and present with headache and seizures. Most of the tumors are located in the supratentorial compartment. The presence of a cyst together with the tumor appears to be characteristic of intraparenchymal schwannoma of the brain [2].

Histopathologically, the detection of Antoni A and Antoni B structures, Verocay bodies, infiltration by foamy macrophages, and vascular hyalinization usually suffice for the recognition of schwannomas. 

 Surgery remains the main therapeutic modality, and due to the benign nature of the tumor, complete excision is associated with cure, and the long-term outcome after excision is generally good. The patient was discharged from our hospital with no neurological deficit.

## 4. Conclusion

 This case is reported here because of the rarity of the lesion, and schwannoma should be considered as differential diagnosis in young patients presenting with solid and cystic intracranial space occupying lesions. Long-term outcome after excision is generally good.
